# Supplementary material for: Quasi‑DNS with chemical kinetics for a single multiple‑injection burner element under stable lean conditions for future gas turbine applications
Source: MethodsX. 2026 Oct 19;17:104218. doi: 10.1016/j.mex.2026.104218 (PMC13718670; doi:10.1016/j.mex.2026.104218)
Supplement: Supplementary file 1 [file mmc1.docx]

Fig. S1	Profiles of representative intermediate and radical species under the same conditions as those in Fig. 4: (a) CO, (b) OH, and (c) CH_2_O. Results obtained using OpenFOAM with the Correa mechanism and Le = 1 are compared with those obtained using Cantera with the Correa mechanism and GRI 3.0.

(a)	(b)

(c)	(d)

Fig. S2	Effects of mesh resolution on the mean and standard deviation of the temperature, heat release rate, CH_4_ mass fraction, and axial velocity along a radial profile 10 mm downstream of the mixing tube exit. The trends are similar to those observed along the centerline (Fig. 6).

(a)	(b)

(c)

Fig. S3	Sensitivity of the centerline time-averaged Quasi-DNS profiles to the averaging duration for the partially premixed case at 𝜙 = 0.45. Profiles of (a) temperature, (b) heat release rate, and (c) methane mass fraction were averaged over nested 4 ms, 8 ms, and 16 ms intervals ending at the same final sampling time. The mixing-tube outlet is located at 𝑥 = 0.
